# Supplementary material for: Participant acceptability of exercise in kidney disease (PACE-KD): a feasibility study protocol in renal transplant recipients
Source: BMJ Open. 2017 Sep 24;7(9):e017494. doi: 10.1136/bmjopen-2017-017494 (PMC5623462; doi:10.1136/bmjopen-2017-017494)
Supplement: Author's manuscript [file bmjopen-2017-017494.draft_revisions.pdf]

BMJ Open is committed to open peer review. As part of this commitment we make the peer review history of every article we publish publicly available.

When an article is published we post the peer reviewers' comments and the authors' responses online. We also post the versions of the paper that were used during peer review. These are the versions that the peer review comments apply to.

The versions of the paper that follow are the versions that were submitted during the peer review process. They are not the versions of record or the final published versions. They should not be cited or distributed as the published version of this manuscript.

BMJ Open is an open access journal and the full, final, typeset and author-corrected version of record of the manuscript is available on our site with no access controls, subscription charges or pay-per-view fees (http://bmjopen.bmj.com).

If you have any questions on BMJ Open's open peer review process please email editorial.bmjopen@bmj.com

BMJ Open

Participant Acceptability of Exercise in Kidney Disease (PACE-KD): A feasibility study protocol in renal transplant recipients

Journal:	*BMJ Open*
Manuscript ID	bmjopen-2017-017494
Article Type:	Protocol
Date Submitted by the Author:	25-Apr-2017
Complete List of Authors:	Bishop , Nicolette ; Loughborough University, School of Sport, Exercise and Health Sciences<br>Billany, Roseanne; Leicester Kidney Exercise Team,<br>Smith, Alice; University of Leicester , Department of Infection, Immunity and Inflammation
**Primary Subject Heading**:	Sports and exercise medicine
Secondary Subject Heading:	Cardiovascular medicine, Renal medicine
Keywords:	cardiovascular disease, high intensity interval training, inflammation, IMMUNOLOGY, exercise

SCHOLARONE™
Manuscripts

For peer review only

Participant Acceptability of Exercise in Kidney Disease (PACE-KD): A feasibility study protocol in renal transplant recipients

Authors: Nicolette C Bishop[1], Roseanne Billany[1, 3], Alice C Smith[2,3]

Corresponding author:

Roseanne Billany
Leicester Kidney Exercise Team
Academic Unit
Leicester General Hospital
LE5 4PW
r.billany@lboro.ac.uk

[1] School of Sport, Exercise and Health Sciences, Loughborough University, Loughborough, United Kingdom

[2] Department of Infection, Immunity and Inflammation, University of Leicester, Leicester, United Kingdom

[3] John Walls Renal Unit, University Hospitals of Leicester NHS Trust, Leicester, UK

Keywords: renal transplantation, cardiovascular disease, feasibility, exercise, high intensity interval training, immunology, inflammation

Word count (excluding title page, abstract, references, figures and tables): 4086

For peer review only

For peer review only - http://bmjopen.bmj.com/site/about/guidelines.xhtml

Abstract

Introduction: Cardiovascular disease (CVD) is a major cause of mortality in renal transplant recipients (RTRs). General population risk scores for CVD underestimate the risk in chronic kidney disease (CKD) patients suggesting additional non-traditional factors. Renal transplant recipients also exhibit elevated inflammation and impaired immune function. Exercise has a positive impact on these factors in CKD patients but there is a lack of rigorous research in RTRs, particularly surrounding the feasibility and acceptability of high intensity interval training (HIIT) versus moderate intensity continuous training (MICT) in this population. This study aims to explore the feasibility of three different supervised aerobic exercise programmes in RTRs to guide the design of future large scale efficacy studies.

Methods and Analysis: Renal transplant recipients will be randomised to HIIT A (16 min interval training with 4, 2 and 1 minute intervals at 80-90% VO_2 peak), HIIT B (4 x 4 min interval training at 80-90% VO_2 peak) or MICT (~40 min cycling at 50-60% VO_2 peak) where they will undertake 24 supervised sessions (approximately thrice weekly over 8 weeks). Assessment visits will be at baseline, mid-training, immediate post-training and 3 months post-training. The study will evaluate the feasibility of recruitment, randomisation, retention, assessment procedures and the implementation of the interventions. A further qualitative sub-study (QPACE-KD) will explore patient experiences and perspectives through semi-structured interviews and focus groups.

Ethics and Dissemination: All required ethical and regulatory approvals have been obtained. Findings will be disseminated through conference presentations, public platforms and academic publications.

Trial Registration Number: Prospectively registered; ISRCTN17122775.

Strengths and limitations of this study
• This study provides a multidisciplinary approach with a variety of quantitative and qualitative outcome measures which will contribute to future large scale randomised control trial design with focus on negative CVD outcomes in RTRs<br>• This study addresses the urgent need to determine the feasibility of differing aerobic exercise types in RTRs to inform future advice and recommendations<br>• This study specifically contributes to the limited literature on the feasibility of high intensity interval training in solid organ transplant patients<br>• As a limitation, the sample size is limited due to the study being centred on feasibility in preparation for future efficacy trials

INTRODUCTION

Cardiovascular disease (CVD) is a major cause of morbidity and mortality after kidney transplantation and a key factor limiting graft survival.[1 2] Most recent data suggests that the proportion of mortalities in kidney transplant recipients attributed to CVD or stroke is 22%[3] however, this figure does not emphasise the total impact on graft survival and quality of life of non-fatal events such as acute myocardial infarction, heart failure, stroke and cardiac arrhythmias.[2] Numbers of transplants per year are currently rising,[3] highlighting the need for cost-effective CVD prevention and risk factor management strategies for sustained patient health, graft survival and reduced health care burden.[4]

Traditional models of predicting CVD risk underestimate the risk in chronic kidney disease (CKD) patients suggesting that kidney disease and transplantation introduce unique CVD risk factors not

For peer review only - http://bmjopen.bmj.com/site/about/guidelines.xhtml

akin to the general population.[5] Immunosuppressive medication can exacerbate traditional risk factors such as poor lipid profile, elevated blood pressure and the incidence of new onset diabetes.[6 7] Weight gain, predominantly fat, is common in RTRs with 60% having a mid-arm fat area that is within the top 10% of values for the general population.[8] Unique haemodynamic challenges (anaemia, hypertension and volume expansion) in RTRs can accelerate cardiomyopathy in the absence of the usual observation of concurrent ischemic heart disease.[2] These haemodynamic stresses are a likely contributor to the paradoxically common vascular and systemic inflammation observed in RTRs.[9] Studies have suggested that elevations in novel biomarkers of inflammation are not just indicative but also contributory to CVD development.[10 11]

Empirical evidence suggests that physical activity has 'anti-inflammatory' consequences.[11] Recent exercise intervention studies in kidney patients who have not reached end-stage renal disease (ESRD) demonstrate reduced traditional and novel markers of CVD risk and improved physical function.[12-14] Similarly, dialysis patients have shown lowered markers of systemic inflammation[15] and improvements in arterial stiffness.[16] However, given its probable benefits, there is a shortfall in literature surrounding the role of physical activity in the management of CVD risk in RTRs.[17] With a precarious balance of immune function and non-traditional CVD risk factors, current exercise guidelines may not be appropriate for this population.

Moderate intensity continuous training (MICT) such as 30-40 minutes brisk walking on three days of the week[12] or intradialytic cycling[15] have been utilised and shown to be beneficial in reducing markers of CVD risk in kidney disease patients. There has been a recent abundance of literature surrounding high intensity aerobic protocols, particularly high intensity intermittent training (HIIT) which suggests a greater improvement in traditional and inflammatory markers of CVD risk in clinical and non-clinical populations when compared to MICT.[18] Although protocols differ, HIIT is characterised by short bursts of vigorous exercise (upwards of 80% maximal aerobic capacity) interspersed with periods of moderate intensity exercise or rest. In patients with CVD and heart transplant recipients, findings indicate that HIIT is well tolerated, safe and effective in improving CVD risk.[19-21] Most recently, HIIT has been shown to be effective in improving physical function, inflammation and quality of life in peritoneal dialysis patients[22] however, the effectiveness of HIIT versus MICT in RTRs has not yet, to our knowledge, been evaluated. With a lower time commitment and the aforementioned superior benefits, HIIT has the potentially to provide a creditable alternative to MICT. This type of exercise has also been perceived to be more enjoyable than MICT,[23] a key component for changing and maintaining physical activity levels in RTRs identified through qualitative methods.[24] However, our patients' views suggest that although HIIT may provide superior improvements of CVD risk factors with a shorter time commitment, the strenuous nature of the exercise might in itself present a barrier to participation in this population[24], and MICT such as brisk walking, may be more acceptable and encourage sustained participation.

Considering both the development of effective and sustainable exercise guidelines for lowering the CVD risk in RTRs and producing programmes with a wide appeal and good adherence, this pilot research aims to generate substantial foundations towards understanding the feasibility and acceptability of differing aerobic exercise protocols in this unique population. The finding will contribute to the growing body of information not only in this population but in other chronic disease populations at risk of CVD and also determine the feasibility of proceeding to large and complex randomised control trials with primary outcomes focusing on the reduction of negative CVD outcomes.

Objectives

Primary Objective

For peer review only - http://bmjopen.bmj.com/site/about/guidelines.xhtml

To evaluate the feasibility of a trial protocol involving three different supervised aerobic exercise programmes in renal transplant recipients.

Secondary Objectives

1. To assess participant experience and acceptability.
2. To explore potential for impact on physical function, body composition, habitual physical activity and non-invasive CVD risk factors.
3. To explore potential for impact on relevant blood, saliva and urinary markers of metabolic health, systemic inflammatory status and immune function.
4. To assess the tolerability, practicality and patient perceived usefulness of these assessments of cardiovascular disease risk.
5. To explore patient perception of their illness and symptoms, activity levels and quality of life.

METHODS AND ANALYSIS

PACE-KD Trial Design

The PACE-KD trial is a randomised, three-arm parallel-group study to determine the feasibility and acceptability of three different supervised aerobic exercise programmes. Figure 1 presents the study flow chart. There will be three parallel groups (exercise programmes): HIIT intervention A, HIIT intervention B and MICT (detailed below). Participants will be randomised to one of the three study arms and will complete 24 supervised sessions of the intervention (approximately three sessions per week over eight weeks). After initial baseline testing, outcome measures will be attained at mid-training, immediate post training and three months post training. Additional acute outcome measures will be taken during one exercise session in the first six weeks and during one exercise session in the last six weeks of each exercise programme (See Figure 2). Feasibility will be determined by assessing recruitment, compliance and completion. Acceptability and patient experience will be determined by a Patient Satisfaction Questionnaire (PSQ) and an optional nested qualitative study (QPACE-KD) consisting of either one to one semi-structured interviews or focus groups.

Patient Involvement

The study was designed in consultation with patient partners. A patient involvement (PPI) group has been convened (lead by JS) and will meet regularly with the rest of the research team to review progress and address any issues that arise. The PPI partners will particularly take part in the qualitative analysis, and interpretation and dissemination of the results.

Participants and Recruitment

A total of 24 participants with an established kidney transplant will be randomised (via computerised random number generator) to either HIIT intervention A, HIIT intervention B or MICT (see Table 1 for detailed inclusion and exclusion criteria). Patients will be screened by their own clinician for eligibility to enter the study. Eligible patients will be approached during their routine clinical appointments and will be provided with verbal and written study information. Additionally, eligible patients who have given prior consent to be contacted regarding research opportunities will be contacted via the post. All patients will be given the opportunity to discuss the study in more detail and the opportunity to consider their participation (at least 48 hours).

For peer review only - http://bmjopen.bmj.com/site/about/guidelines.xhtml

Table 1. PACE-KD Inclusion and Exclusion Criteria

Inclusion Criteria	Exclusion Criteria
• Diagnosed with CKD and are a renal transplant recipient<br>• Male or female, aged 18 years or over<br>• Received renal transplant > 12 weeks prior to entering the study<br>• Is willing and able to give informed consent for participation in the study	• Aged under 18 years<br>• Female participants who are pregnant, lactating, or planning pregnancy during the course of the study<br>• Scheduled elective surgery or other procedures requiring general anaesthesia during the study<br>• Any other significant disease or disorder*<br>• Inability to give informed consent or comply with testing and training protocol for any reason

*i.e. significant co-morbidity including unstable hypertension, potentially lethal arrhythmia, myocardial infarction within 6 months, unstable angina, active liver disease, uncontrolled diabetes mellitus (HbA1c ≥ 9%), advanced cerebral or peripheral vascular disease which, in the opinion of the patient's own clinician, may either put the patient at risk because of participation in the study, or may influence the result of the study, or the patient's ability to participate in the study.

Interventions

Each exercise programme will consist of 24 sessions over approximately 8 weeks. Participants will be allowed to drink plain water during exercise sessions. The programmes will be based on the three protocols described below.

HIIT programme A: (Figure 3a)

Each session will consist of 16-min interval training with intervals of 4, 2 and 1 min duration at 80%, progressing to 90% $VO_{2\ peak}$ over the 8 weeks, separated by a 2 min active rest (~60% $VO_{2\ peak}$) giving a total exercise time of 30 min.

HIIT programme B: (Figure 3b)

Each supervised session will consist of 4 x 4-min of interval training at 80% $VO_{2\ peak}$, progressing to 90% $VO_{2\ peak}$ over the 8 weeks, separated by a 3 min active rest (~60% $VO_{2\ peak}$) and with a final 5 min recovery stage to ensure that overall work done is matched for both HIIT protocols, therefore also giving a total exercise time of 30 min.

MICT Exercise Intervention

Each supervised session will consist of continuous brisk cycling for ~40 min with a target RPE of 12-14 (somewhat hard). This is equivalent to 50-60% $VO_{2\ peak}$ and will be matched to the HIIT protocols for total external work done based on calculations of percentage of peak watts obtained from the $VO_{2\ peak}$ test. In this way, for each individual the overall energy expenditure during the session would be the same regardless of the trial they are assigned to.

All exercise sessions will be preceded by a 5 min low intensity warm up and followed by a 10 min cool down.

For peer review only - http://bmjopen.bmj.com/site/about/guidelines.xhtml

Study Procedures

The study outcomes, measurement methods and assessment time periods are summarised in Table 2 and a detailed overview of each measurement is provided at the end of this section. Data on recruitment, numbers of sessions attended and completed in accordance with the protocol and drop out will be recorded and continuously monitored.

Table 2. PACE-KD outcomes, measurement methods and assessment time periods

Outcome	Measurement Method/Tool	Time Period
Feasibility	Recruitment, compliance, drop-out and completion percentages	Continuous
$VO_{2\ peak}$	CPET	Baseline, post-training
Physical Function	Gait speed test<br>Sit to stand test<br>Plantar flexion Strength (Fysiometer)	Baseline, mid-training, post-training, 3M post-training
Body Composition	BIA	Baseline, mid-training, post-training, 3M post-training
Heart Function	NICOM	Baseline, mid-training, post-training, 3M post-training
Arterial Stiffness	PWV (Applanation Tonometry)	Baseline, mid-training, post-training, 3M post-training
SpO_2	Pulse Oximetry	Baseline, mid-training, post-training, 3M post-training, acute response sessions
Habitual Physical Activity	Accelerometry	Baseline, post-training
Muscle O_2 Saturation	NIRS	Acute response session
Urinary Bacteriuria	Urine Sample	Baseline, mid-training, post-training
Immune Function	Blood and Saliva Samples	Baseline, mid-training, post-training, 3M post-training, acute response sessions
Markers of inflammation, oxidative stress, CVD risk	Blood Samples	Baseline, mid-training, post-training, 3M post-training, acute response sessions
Renal Profile	Blood Samples	Baseline, post training
Clinical Information	Extraction from clinical notes	Baseline, mid-training, post-training, 3M post-training
Renal Symptoms	POS-S	Baseline, mid-training, post-training, 3M post-training
Fatigue	Fatigue Scale	Baseline, mid-training, post-training, 3M post-training
Quality of Life	EQ-5D	Baseline, mid-training, post-training, 3M post-training
Illness Perception	IPQ-R	Baseline, mid-training, post-training, 3M post-training
Physical Activity/ Functional Capacity	DASI	Baseline, mid-training, post-training, 3M post-training
Pain	BPI	Baseline, mid-training, post-training, 3M post-training
Sleep Quality	PSQI	Baseline, mid-training, post-training, 3M post-training
Daytime Sleepiness	ESS	Baseline, mid-training, post-training, 3M post-training
Illness Symptoms	PACE-KD Illness-Symptom Questionnaire	Before each exercise session and x1 at the weekend between sessions
Tolerability, practicality, acceptability and perceived usefulness of the study measures	PSQ;<br>Semi-structured interview or focus group	Post study involvement;<br>Post study involvement through optional QPACE-KD nested qualitative study

Abbreviations: $VO_{2\ peak}$, Peak Oxygen Uptake; CPET, Cardiopulmonary Exercise Test; BIA, Bioelectrical Impedance Analysis; NICOM, Non-Invasive Cardiac Output Monitor; SpO_2, Peripheral Oxygen Saturation; NIRS, Near-infrared Spectroscopy; CVD, Cardiovascular Disease; POS-S, Renal Patient Outcome Scale; EQ-5D, EuroQol; IPQ-R, Illness Perception Questionnaire - Revised; DASI, Duke Activity Status Index; BPI, Brief Pain Inventory; PSQI, Pittsburgh Sleep Quality Index; ESS, Epworth Sleepiness Scale; PSQ, Patient Satisfaction Questionnaire

Baseline Visit (Visit 1)

At visit one informed consent will be obtained on arrival. Participants will perform a standard maximal exercise test ($VO_{2\ peak}$ test) in the presence of a cardiac nurse. This test will be preceded by a 12-lead electrocardiogram (ECG) and subject to the confirmed absence of any contraindications.

For peer review only - http://bmjopen.bmj.com/site/about/guidelines.xhtml

Measurements of cardiac function, physical function, anthropometry, body composition and blood, urine and saliva samples will be collected. A survey pack will be completed and an accelerometer given to measure habitual physical activity over approximately 7 days.

Training visits

Participants will attend 24 supervised exercise sessions (approximately 3 per week for 8 weeks) of either: HIIT A, HIIT B or MICT (as outlined above). At the beginning of each session participants will be asked to complete an internally designed Illness-Symptom questionnaire. They will also be provided with a copy to complete over the weekend between sessions. The questionnaire will provide data on illness symptoms particularly relating to upper respiratory, digestive and urinary tracts.

Acute Response Sessions

At one of the first six and at one of the final six exercise sessions, a resting blood sample will be collected immediately prior to the usual exercise session, immediately post exercise session, 1 h post exercise session, 4 h post exercise session and immediately prior to the start of the next exercise session. Saliva samples will be taken in conjunction with each blood sample. Participants will be asked to abstain from caffeine and alcohol for 24 h prior to these two acute testing sessions, and on acute testing session 2 asked to consume a similar diet to the 24 h prior to acute session 1. During the exercise session a muscle oxygen saturation monitor will be attached to the calf muscle (non-invasively) and will be removed following the 4 h blood sample. Following the 1 h samples, prior to the 4 h sample, patients will be provided with light refreshments. They will be asked to refrain from consuming alcohol and caffeine and from partaking in strenuous exercise.

Mid-training Visit

The mid-training visit will take place between weeks 3 and 5. At this visit, the experimental tests described in the baseline visit will be repeated with the exception of the $VO_{2\ peak}$ test and the accelerometer for habitual physical activity. The survey pack as described in the baseline visit will be administered, completed and collected during this visit.

Immediate Post-training visit

The immediate post-training visit will take place within 14 days of the final exercise session taking place. All experimental tests and the survey pack outlined in the baseline visit will be repeated. An accelerometer will be fitted as in the baseline visit and returned after one week.

Three Month Post-Training Visit

The three month post-training visit will take place approximately three months following the final exercise session. The visit will be very similar to the mid-training visit. All experimental tests and the survey pack outlined in the aforementioned visit will be repeated with the exclusion of the $VO_{2\ peak}$ test. There will be no more experimental measures after this visit.

An internally designed Patient Satisfaction Questionnaire (PSQ) will be administered once at the end of study participation. The PSQ is designed to assess the acceptability of the study and experiences of the patients in each of the trial groups with particular focus on tolerability, practicality, enjoyment and perceived usefulness of the study measures and of the study as a whole. Participants who cease

For peer review only - http://bmjopen.bmj.com/site/about/guidelines.xhtml

involvement in the study prior the final assessments will also be invited to complete the questionnaire. This will be optional.

Study Outcome Measures

$VO_{2\ peak}$ Test

Standard ramp protocol; increasing workload of approximately 1 Watt every 4 sec (10-15 w/min) ensuring volitional exhaustion within 12-15 min[25] on a stationary electronically braked cycle ergometer. Participants will be encouraged to cycle at a continuous cadence (~70 rpm). The highest oxygen uptake will be measured in this study ($VO_{2\ peak}$) as true maximal oxygen uptake ($VO_{2\ max}$) is less commonly achieved in deconditioned and/or clinical patients. Results of this test will be used to determine training workloads.

Clinical information

Clinical information will be extracted from the medical notes including: age, gender, ethnicity, primary cause of renal failure, transplant type, time since transplant, dialysis duration, co-morbidities, blood/urine results, current medication and smoking habits. This information will be used to primarily account for cofounding variables and analyses of differences and similarities between intervention arm groups.

Anthropometric measures

Anthropometric measures of height, weight and waist and hip circumference will be attained in accordance with standard protocols.[26]

Body Composition

Bioelectrical impedance analysis (BIA) performed on an InBody analyser (InBody 370, Chicago, IL, USA) will be used to assess body composition (e.g. body fat percentage and fat free mass). This is a painless, non-invasive method[27 28] previously validated for use in CKD patients.[29]

Cardiovascular Condition and Function

A Non-Invasive Cardiac Output Monitor (NICOM; Cheetah Medical, Maidenhead, UK) will be used to assess heart function using cardiac bioreactance analysis. This method is safe, quick and validated[30] for assessing haemodynamic parameters including cardiac output (heart rate x stroke volume) and total peripheral resistance.

Pulse Wave Analysis (PWA)

Pulse wave analysis will be measured non-invasively to determine arterial stiffness using applanation tonometry methods (Sphygmocor, AtCor Medical Pty Ltd, West Ryde, NSW, Australia). Arterial pressure waveforms are recorded superficially at the radial artery by an experienced laboratory technician by applanating the artery with a high-fidelity hand-held tonometer at the site of maximal pulsation. Consistent and reproducible waveforms are recorded for 20 seconds. This is a safe procedure and a measure of this type has been performed in CKD patients previously.[13 14]

Physical Function

Physical function will be assessed using three tests. The Sit-to-stand (STS) test will be used to ascertained functional ability and has been widely used in the CKD population.[31-33] Participants will

For peer review only - http://bmjopen.bmj.com/site/about/guidelines.xhtml

be asked to sit on a hard upright chair with their feet flat on the floor and knees bent at 90°. One cycle is considered complete when the participant stands up and sits down again to the starting position (without using their hands). There are two tests within the STS:

- *STS5*: The time taken for patients to stand up from a seated position and sit back down again 5 times. It is a surrogate measure of muscular power.

- *STS60* : The number of STS cycles achieved in 60 seconds and is a surrogate measure of muscular endurance.

The 4 meter walk test will be used to assess gait speed. Participants will be asked to walk 4 meters at their usual pace for one practice and two times trials. The better score (in sec) on the timed trials will be recorded.

Isometric plantar flexion strength (gastrocnemius and soleus strength) will be assessed using a Wii Fit board (Nintendo, Kyoto, Japan). Participants will sit with their legs bent to 90° with their feet flat on the board. A strap will be placed around the board and over the knees. Participants will then be asked to raise their heels off the board causing a downward force to be exerted on the board.

Muscle Oxygen Saturation

Prior to the acute testing sessions a small non-invasive muscle oxygen saturation device (BSXInsight, BSXAthletics, TX, USA) will be placed onto the calf muscle and secured with a small bandage. The device will be removed following the 4 h sample collection. This will measure muscle oxygen saturation using near infrared spectroscopy (NIRS) and has been used in a variety of diseases previously.[34]

Saliva sampling and storage

Saliva samples will be collected into sterile plastic containers. Participants will swallow to empty the mouth, then open and hold the container themselves before performing a passive dribble of saliva collected under the tongue over the next two minutes. Following centrifugation, the supernatant will be aliquoted and frozen for future analysis, primarily for secretory Immunoglobulin A (sIgA) to investigate mucosal immunity.

Urine Sampling and Storage

A urine sample will be requested during the baseline visit. This sample will be analysed for urinary protein excretion, bacteriuria and urinary tract infection susceptibility.

Venous Blood Sampling and Analysis

Venous blood will be collected using venepuncture of the antecubital vein and prepared appropriately for the following analysis:

- A cytometric bead array technique will be used to allow bulk-analysis of a panel of pro- and anti-inflammatory cytokines, including but not limited to IL-1, IL-2, IL-6, IL-10, TNF-α and IFNγ.
- Other markers of inflammation (CRP) and oxidative stress (MDA) and will be assessed by ELISA.

For peer review only - http://bmjopen.bmj.com/site/about/guidelines.xhtml

- Phenotyping of microparticles using flow cytometry.
- Immune cell subsets using flow cytometry.
- Full blood count and renal profile analysis, which includes eGFR, urea, bicarbonate, creatinine, sodium, potassium and phosphate measures.

Questionnaires

Participants will be asked to complete a survey pack containing the following: (1) Renal Patient Outcome Scale (POS-S); a validated questionnaire that measures the presence and severity of disease related symptoms with the addition of a transplant specific question set.[35] (2) Fatigue Scale; an internally designed Likert scale on feelings of tiredness. (3) EuroQol (EQ-5D); a short widely used quality of life questionnaire.[36] (4) Illness Perception Questionnaire – Revised (IPQ-R); a 84 item questionnaire which is used to assess the principle components of illness representations.[37] This questionnaire has been tailored for patients with chronic kidney disease and will assess their perceptions of physical activity as treatment. (5) Duke Activity Status Index (DASI); a 12-item questionnaire that utilizes self-reported physical work capacity to estimate peak metabolic equivalents (METs) and has been shown to be a valid measurement of functional capacity.[38] (6) Brief Pain Inventory – Short Form (BPI-sf); a 9 item self-administered questionnaire used to evaluate the severity of a patient's pain and the impact of this pain on the patient's daily functioning.[39]. (7) The Pittsburgh Sleep Quality Index (PSQI); a self-report questionnaire that assesses sleep quality over a 1-month time interval[40] (8) Epworth Sleepiness Scale (ESS); the ESS measures a person's general level of daytime sleepiness, or their average sleep propensity in daily life (ASP). [41]

Accelerometry

An ActiGraph wGT3X-BT accelerometer (Actigraph, Pensacola, Florida, USA) will be provided to wear on the waist which will record habitual physical activity (returned after one week).

Nested Qualitative Study (QPACE-KD)

Six participants from each intervention arm will be invited to an audio-recorded focus group or semi-structured interview to discuss their experiences of the study and the intervention in more detail and to gather suggestions for relevant and acceptable design of future randomised controlled trials. The qualitative phase will be conducted by a practiced researcher uninvolved with the aforementioned study protocol to encourage freedom of expression. Both one-to-one semi-structured interviews and focus groups will be used to provide different but complementary qualitative information. The interviews will ascertain individual perspectives and feelings about the intervention and outcome protocol in the light of personal and individual experiences of having completed the feasibility study. On the other hand, focus groups will elicit perceptions which are particularly useful when there is some consensus and meaning about the existence of a community or shared voice.

The QPACE-KD study is an optional addition to PACE-KD. The QPACE-KD protocol will be explained to the patient, and they will be given ample opportunity to ask questions and at least 48 hours to decide whether or not to participate. If they agree, separate QPACE-KD written informed consent will be attained before the interview or focus group takes place. During the interviews and focus groups, the facilitator will introduce the process, explaining the background and reasons for the study, how the interview will proceed, and details of audio-recording and note-taking. Participants will be reassured about the preservation of their anonymity and confidentiality as well as given the opportunity to ask questions.

For peer review only - http://bmjopen.bmj.com/site/about/guidelines.xhtml

Transcripts will only include non-identifying information. Individual's names and personal details will not be included in the completed transcripts. Similarly, any written notes taken during the interviews will not include any identifiable personal data.

Data Collection and Management

Data at all time points will be collected in case report forms by the trial team. All data will be entered into a secure database and will only be accessible on password protected computers at University Hospitals of Leicester NHS Trust, University of Leicester and Loughborough University by relevant members of the study team. No identifying information will be kept in electronic form. All source data and original participant identities will be kept in a locked office in the trial site file only at University Hospitals of Leicester NHS Trust.

Sample Size

As the trial has a primary outcome of feasibility the study sample size is pragmatic and a power calculation is neither relevant nor possible. Data collected on secondary outcome measures will provide data on which sample size calculations can be performed in future well informed randomised controlled trials. We have chosen specific and relevant secondary outcome variables that may be suitable future primary outcomes.

Data Analysis

The primary outcome will assess the feasibility of study components such as recruitment, randomisation, retention, assessment procedures and the implementation of the interventions. These will be quantified by analysing factors such as but not limited to: numbers screened, numbers enrolled, proportion of eligible patients enrolled, intervention-specific retention rates, rates of adherence to protocol per intervention and proportion of training sessions and assessment sessions completed.

Interview data will be analysed according to the principles of interpretive thematic analysis using a framework approach[42] to explore themes emerging from patient journeys through and experiences of the interventions and outcome measures. The whole research team, including patient partners, will be involved in qualitative analysis to avoid individual bias.

Ethics and Dissemination

Ethical issues

The University Hospitals of Leicester NHS Trust (UHL) agreed to act as sponsor for this study on 31/10/2016 (EDGE 88714). The protocol was reviewed by the East Midlands-Nottingham Research Ethics Committee (REC) and was given a favourable opinion (REC ref 16/EM/0482) on 04/01/2017. Health Research Authority regulatory approval was given on 27/01/2017, and the study was adopted on the NIHR portfolio on 12/01/2017. Local governance approval was granted by UHL Research and Innovation on 02/03/2017. Steps have been taken when designing this protocol to minimise the ethical implications and ensure patient welfare. The study will comply with the International Conference for Harmonisation of Good Clinical Practice (ICH GCP) guidelines and the Research Governance Framework for Health and Social Care.

Dissemination Plan and Impact

For peer review only - http://bmjopen.bmj.com/site/about/guidelines.xhtml

On completion the results of this study will be published in peer-reviewed journals and presented at national and international conferences. Participant level data will be available following publication of results on request. Results will also be disseminated to the patient and public community via social media and newsletter articles and presentations at patient conferences and forums, lead by the patient partners.

It is anticipated that the results of this study will inform future design of larger randomised controlled trials in this subject area and contribute to future specific physical activity guidelines in this population.

Trial registration

This study was prospectively registered with the ISRCTN (ISRCTN17122775).

Twitter: Follow Roseanne Billany @RBillany

Contributors: ACS is the chief investigator for this trial. ACS and NCB lead on study design and protocol preparation. RB wrote this manuscript and contributed to protocol preparation, ethics application and document preparation. All authors reviewed and approved the final version of this manuscript.

Funding: This study is jointly funded by Heart Research UK grant number RG2650/15/18, the Stoneygate Trust and the Engineering and Physical Sciences Research Council (EPSRC) Antimicrobial Resistance (AMR) grant. The research was supported by the National Institute for Health Research (NIHR) Leicester Biomedical Research Centre. The views expressed are those of the authors and not necessarily those of the NHS, the NIHR or the Department of Health.

Acknowledgements: We would like to thank Dr Clare Stevinson (Loughborough University), Dr James O Burton (University Hospitals of Leicester NHS Trust) and Mr John Savage for their valuable work towards the development of the funding application for this study.

Competing Interests: None declared.

Figure legends:

Figure 1. Study Flowchart: Overall study diagram.

Figure 2. Study Flowchart: Acute response sessions detailed diagram.

Figure 3. Schematic diagram of HIIT regimens A and B. Each block shows stage duration in min. The final 5 minute stage has been added to HIIT programme B to ensure the overall work done is matched for both protocols.

References

1. Chan W, Bosch JA, Jones D, et al. Obesity in kidney transplantation. *J Ren Nutr* 2014;24(1):1-12.
2. Kiberd BA. Cardiovascular Disease in Kidney Transplant Recipients. *Adv Stud Med* 2007;7(6):169-78.

For peer review only - http://bmjopen.bmj.com/site/about/guidelines.xhtml

3. Pruthi R, Casula A, MacPhee I. UK Renal Registry 17th Annual Report: Chapter 3 Demographic and Biochemistry Profile of Kidney Transplant Recipients in the UK in 2013: National and Centre-specific Analyses. *Nephron* 2015;129 Suppl 1:57-86.
4. Mathur AK, Chang Y-H, Steidley ED, et al. Patterns of Care and Outcomes in Cardiovascular Disease After Kidney Transplantation in the United States. *Transplant Direct* 2017;3(2):e26.
5. Mansell H, Rosaasen N, Dean J, et al. Evidence of enhanced systemic inflammation in stable kidney transplant recipients with low Framingham risk scores. *Clin Transplant* 2013;27(4):E391-9.
6. Boots JM, Christiaans MH, van Hooff JP. Effect of immunosuppressive agents on long-term survival of renal transplant recipients: focus on the cardiovascular risk. *Drugs* 2004;64(18):2047-73.
7. Heisel O, Heisel R, Balshaw R, et al. New onset diabetes mellitus in patients receiving calcineurin inhibitors: a systematic review and meta-analysis. *Am J Transplant* 2004;4(4):583-95.
8. Schutz T, Hudjetz H, Roske AE, et al. Weight gain in long-term survivors of kidney or liver transplantation - another paradigm of sarcopenic obesity? *Nutrition* 2012;28(4):378-83.
9. Abedini S, Holme I, Marz W, et al. Inflammation in renal transplantation. *Clin J Am Soc Nephrol* 2009;4(7):1246-54.
10. Kasapis C, Thompson PD. The effects of physical activity on serum C-reactive protein and inflammatory markers: a systematic review. *J Am Coll Cardiol* 2005;45(10):1563-69.
11. Gleeson M, Bishop NC, Stensel DJ, et al. The anti-inflammatory effects of exercise: mechanisms and implications for the prevention and treatment of disease. *Nat Rev Immunol* 2011;11(9):607-15.
12. Viana JL, Kosmadakis GC, Watson EL, et al. Evidence for anti-inflammatory effects of exercise in CKD. *J Am Soc Nephrol* 2014;25(9):2121-30.
13. Mustata S, Groeneveld S, Davidson W, et al. Effects of exercise training on physical impairment, arterial stiffness and health-related quality of life in patients with chronic kidney disease: a pilot study. *Int J Nephrol Urol* 2011;43(4):1133-41.
14. Kosmadakis GC, John SG, Clapp EL, et al. Benefits of regular walking exercise in advanced pre-dialysis chronic kidney disease. *Nephrol Dial Transplant* 2012;27(3):997-1004.
15. Dungey M, Bishop NC, Young HML, et al. A Six-Month Intradialytic Exercise Programme Has Anti-Inflammatory Effects on Circulating Monocyte Phenotypes and Regulatory T Cells But Not on Cytokine Concentration. 52nd ERA-EDTA Congress. London, 2015.
16. Mustata S, Chan C, Lai V, et al. Impact of an exercise program on arterial stiffness and insulin resistance in hemodialysis patients. *J Am Soc Nephrol* 2004;15(10):2713-18.
17. Didsbury M, McGee RG, Tong A, et al. Exercise training in solid organ transplant recipients: a systematic review and meta-analysis. *Transplantation* 2013;95(5):679-87.
18. Ramos JS, Dalleck LC, Tjonna AE, et al. The impact of high-intensity interval training versus moderate-intensity continuous training on vascular function: a systematic review and meta-analysis. *Sports Med* 2015;45(5):679-92.
19. Rognmo O, Hetland E, Helgerud J, et al. High intensity aerobic interval exercise is superior to moderate intensity exercise for increasing aerobic capacity in patients with coronary artery disease. *Eur J Cardiovasc Prev Rehabil* 2004;11(3):216-22.
20. Wisloff U, Stoylen A, Loennechen JP, et al. Superior cardiovascular effect of aerobic interval training versus moderate continuous training in heart failure patients: a randomized study. *Circulation* 2007;115(24):3086-94.
21. Dall CH, Gustafsson F, Christensen SB, et al. Effect of moderate- versus high-intensity exercise on vascular function, biomarkers and quality of life in heart transplant recipients: A randomized, crossover trial. *J Heart Lung Transplant* 2015;34(8):1033-41.
22. Rouchon MI, Coutard C, Matysiak M et al. High Intensity Interval Training Improves Physical Functioning, Inflammation and Quality of Life in Peritoneal Dialysis Patients. *Nephrol Dial Transplant* 2016;31(suppl_1)

For peer review only

23. Bartlett JD, Close GL, MacLaren DP, et al. High-intensity interval running is perceived to be more enjoyable than moderate-intensity continuous exercise: implications for exercise adherence. *J Sports Sci* 2011;29(6):547-53.
24. Lloyd-Davies LH, Clarke AL, Brown SA, et al. Renal Transplant Recipient Perspectives on Physical Activity and Exercise. Proceedings of the American Society of Nephrology Annual Meeting 2014.
25. Ross R. ATS/ACCP Statement on Cardiopulmonary Exercise Testing. *Am J Respir Crit Care Med* 2003;167(2).
26. Eston R, Reilly T. Kinanthropometry and Exercise Physiology Laboratory Manual: Tests, Procedures and Data. 3 ed. Oxon: Routledge 2009.
27. Lukaski HC, Bolonchuk WW, Hall CB, et al. Validation of tetrapolar bioelectrical impedance method to assess human body composition. *J Appl Physiol* 1986;60(4):1327-32.
28. Macdonald JH, Marcora SM, Jibani M, et al. Bioelectrical impedance can be used to predict muscle mass and hence improve estimation of glomerular filtration rate in non-diabetic patients with chronic kidney disease. *Nephrol Dial Transplant* 2006;21(12):3481-87.
29. Chertow GM, Lowrie EG, Wilmore DW, et al. Nutritional assessment with bioelectrical impedance analysis in maintenance hemodialysis patients. *J Am Soc Nephrol* 1995;6(1):75-81.
30. Squara P, Denjean D, Estagnasie P, et al. Noninvasive cardiac output monitoring (NICOM): a clinical validation. *Intensive Care Med* 2007;33(7):1191-94.
31. Koufaki P, Mercer TH, Naish PF. Effects of exercise training on aerobic and functional capacity of end-stage renal disease patients. *Clin Physiol Funct Imaging* 2002;22(2):115-24.
32. McIntyre CW, Selby NM, Sigrist M, et al. Patients receiving maintenance dialysis have more severe functionally significant skeletal muscle wasting than patients with dialysis-independent chronic kidney disease. *Nephrol Dial Transplant* 2006;21(8):2210-16.
33. Johansen KL, Painter PL, Sakkas GK, et al. Effects of resistance exercise training and nandrolone decanoate on body composition and muscle function among patients who receive hemodialysis: A randomized, controlled trial. *J Am Soc Nephrol* 2006;17(8):2307-14.
34. Grassi B, Quaresima V. Near-infrared spectroscopy and skeletal muscle oxidative function in vivo in health and disease: a review from an exerise physiology perspective. *J Biomed Opt* 2016;21(9)
35. Hearn J, Higginson IJ. Development and validation of a core outcome measure for palliative care: the palliative care outcome scale. Palliative Care Core Audit Project Advisory Group. *Qual Health Care* 1999;8(4):219-27.
36. Herdman M, Gudex C, Lloyd A, et al. Development and preliminary testing of the new five-level version of EQ-5D (EQ-5D-5L). *Qual Life Res* 2011;20(10):1727-36.
37. Moss-Morris R. The Revised Illness Perception Questionnaire (IPQ-R). *Psychol Health* 2002;17(1):1-16.
38. Hlatky MA, Boineau RE, Higginbotham MB, et al. A brief self-administered questionnaire to determine functional capacity (the Duke Activity Status Index). *Am J Cardiol* 1989;64(10):651-54.
39. Cleeland CS, Ryan KM. Pain assessment: global use of the Brief Pain Inventory. *Ann Acad Med Singapore* 1994;23(2):129-38.
40. Buysse DJ, Reynolds CF, 3rd, Monk TH, et al. The Pittsburgh Sleep Quality Index: a new instrument for psychiatric practice and research. *Psychiatry Res* 1989;28(2):193-213.
41. Johns MW. A new method for measuring daytime sleepiness: the Epworth sleepiness scale. *Sleep* 1991;14(6):540-45.
42. Smith J, Firth J. Qualitative data analysis: the framework approach. *Nurse Res* 2011;18(2):52-62.

For peer review only - http://bmjopen.bmj.com/site/about/guidelines.xhtml

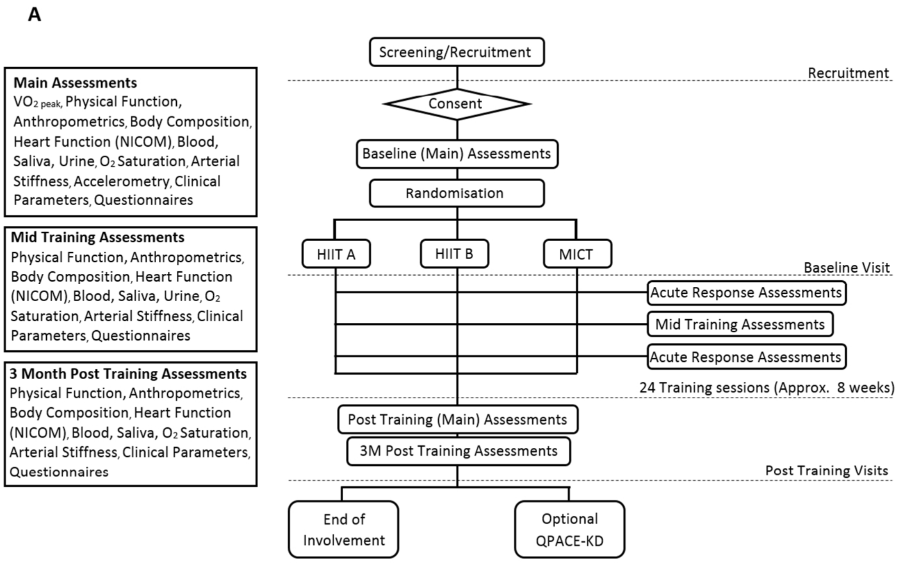

Figure 1. Study Flowchart: Overall study diagram.

384x217mm (96 x 96 DPI)

For peer review only - http://bmjopen.bmj.com/site/about/guidelines.xhtml

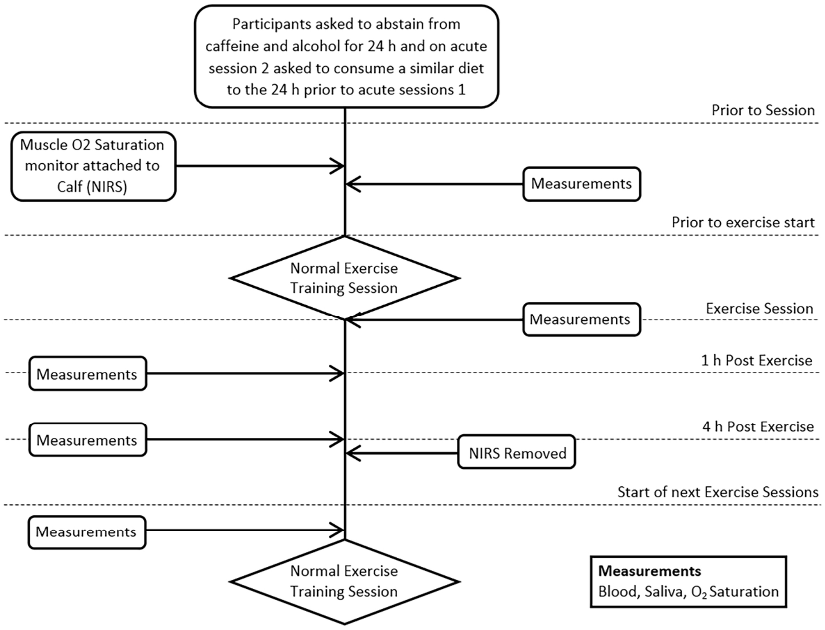

Figure 2. Study Flowchart: Acute response sessions detailed diagram.

101x65mm (600 x 600 DPI)

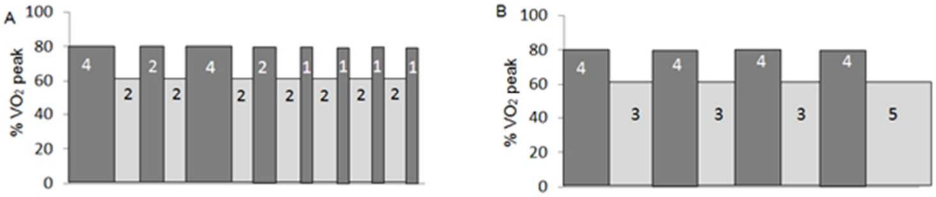

Figure 3. Schematic diagram of HIIT regimens A and B. Each block shows stage duration in min. The final 5 minute stage has been added to HIIT programme B to ensure the overall work done is matched for both protocols.

176x38mm (96 x 96 DPI)

For peer review only

CONSORT 2010 checklist of information to include when reporting a randomised trial*

Section/Topic	Item No	Checklist item	Reported on page No
**Title and abstract**			
	1a	Identification as a randomised trial in the title – not included at it is a feasibility study and protocol paper so space was limited.	x
	1b	Structured summary of trial design, methods, results, and conclusions (for specific guidance see CONSORT for abstracts)	2
**Introduction**			
Background and objectives	2a	Scientific background and explanation of rationale	2-3
	2b	Specific objectives or hypotheses	3-4
**Methods**			
Trial design	3a	Description of trial design (such as parallel, factorial) including allocation ratio	4
	3b	Important changes to methods after trial commencement (such as eligibility criteria), with reasons	N/A
Participants	4a	Eligibility criteria for participants	4-5
	4b	Settings and locations where the data were collected	4
Interventions	5	The interventions for each group with sufficient details to allow replication, including how and when they were actually administered	5
Outcomes	6a	Completely defined pre-specified primary and secondary outcome measures, including how and when they were assessed	6-10
	6b	Any changes to trial outcomes after the trial commenced, with reasons	N/A
Sample size	7a	How sample size was determined	11
	7b	When applicable, explanation of any interim analyses and stopping guidelines	N/A
Randomisation:			
Sequence generation	8a	Method used to generate the random allocation sequence	4
	8b	Type of randomisation; details of any restriction (such as blocking and block size)	4
Allocation concealment mechanism	9	Mechanism used to implement the random allocation sequence (such as sequentially numbered containers), describing any steps taken to conceal the sequence until interventions were assigned	
Implementation	10	Who generated the random allocation sequence, who enrolled participants, and who assigned participants to interventions	4

For peer review only - http://bmjopen.bmj.com/site/about/guidelines.xhtml

Blinding	11a	If done, who was blinded after assignment to interventions (for example, participants, care providers, those assessing outcomes) and how	N/A
	11b	If relevant, description of the similarity of interventions	5
Statistical methods	12a	Statistical methods used to compare groups for primary and secondary outcomes	11
	12b	Methods for additional analyses, such as subgroup analyses and adjusted analyses	11
**Results**			
Participant flow (a diagram is strongly recommended)	13a	For each group, the numbers of participants who were randomly assigned, received intended treatment, and were analysed for the primary outcome	N/A protocol
	13b	For each group, losses and exclusions after randomisation, together with reasons	N/A protocol
Recruitment	14a	Dates defining the periods of recruitment and follow-up	N/A protocol
	14b	Why the trial ended or was stopped	N/A protocol
Baseline data	15	A table showing baseline demographic and clinical characteristics for each group	N/A protocol
Numbers analysed	16	For each group, number of participants (denominator) included in each analysis and whether the analysis was by original assigned groups	N/A protocol
Outcomes and estimation	17a	For each primary and secondary outcome, results for each group, and the estimated effect size and its precision (such as 95% confidence interval)	N/A protocol
	17b	For binary outcomes, presentation of both absolute and relative effect sizes is recommended	N/A protocol
Ancillary analyses	18	Results of any other analyses performed, including subgroup analyses and adjusted analyses, distinguishing pre-specified from exploratory	N/A protocol
Harms	19	All important harms or unintended effects in each group (for specific guidance see CONSORT for harms)	N/A protocol
**Discussion**			
Limitations	20	Trial limitations, addressing sources of potential bias, imprecision, and, if relevant, multiplicity of analyses	N/A protocol
Generalisability	21	Generalisability (external validity, applicability) of the trial findings	N/A protocol
Interpretation	22	Interpretation consistent with results, balancing benefits and harms, and considering other relevant evidence	N/A protocol
**Other information**			
Registration	23	Registration number and name of trial registry	12
Protocol	24	Where the full trial protocol can be accessed, if available	N/A protocol
Funding	25	Sources of funding and other support (such as supply of drugs), role of funders	12

*We strongly recommend reading this statement in conjunction with the CONSORT 2010 Explanation and Elaboration for important clarifications on all the items. If relevant, we also recommend reading CONSORT extensions for cluster randomised trials, non-inferiority and equivalence trials, non-pharmacological treatments, herbal interventions, and pragmatic trials. Additional extensions are forthcoming: for those and for up to date references relevant to this checklist, see www.consort-statement.org.

For peer review only - http://bmjopen.bmj.com/site/about/guidelines.xhtml

BMJ Open

Participant Acceptability of Exercise in Kidney Disease (PACE-KD): A feasibility study protocol in renal transplant recipients

Journal:	*BMJ Open*
Manuscript ID	bmjopen-2017-017494.R1
Article Type:	Protocol
Date Submitted by the Author:	13-Jul-2017
Complete List of Authors:	Bishop , Nicolette ; Loughborough University, School of Sport, Exercise and Health Sciences<br>Billany, Roseanne; Leicester Kidney Exercise Team,<br>Smith, Alice; University of Leicester , Department of Infection, Immunity and Inflammation
**Primary Subject Heading**:	Sports and exercise medicine
Secondary Subject Heading:	Cardiovascular medicine, Renal medicine
Keywords:	cardiovascular disease, high intensity interval training, inflammation, IMMUNOLOGY, exercise

SCHOLARONE™ Manuscripts

For peer review only

Participant Acceptability of Exercise in Kidney Disease (PACE-KD): A feasibility study protocol in renal transplant recipients

Authors: Nicolette C Bishop[1], Roseanne Billany[1, 3], Alice C Smith[2,3]

Corresponding author:

Roseanne Billany
Leicester Kidney Exercise Team
Academic Unit
Leicester General Hospital
LE5 4PW
r.billany@lboro.ac.uk

[1] School of Sport, Exercise and Health Sciences, Loughborough University, Loughborough, United Kingdom

[2] Department of Infection, Immunity and Inflammation, University of Leicester, Leicester, United Kingdom

[3] John Walls Renal Unit, University Hospitals of Leicester NHS Trust, Leicester, UK

For peer review only

Keywords: renal transplantation, cardiovascular disease, feasibility, exercise, high intensity interval training, immunology, inflammation

Word count (excluding title page, abstract, references, figures and tables): 4086

For peer review only - http://bmjopen.bmj.com/site/about/guidelines.xhtml

Abstract

Introduction: Cardiovascular disease (CVD) is a major cause of mortality in renal transplant recipients (RTRs). General population risk scores for CVD underestimate the risk in chronic kidney disease (CKD) patients suggesting additional non-traditional factors. Renal transplant recipients also exhibit elevated inflammation and impaired immune function. Exercise has a positive impact on these factors in CKD patients but there is a lack of rigorous research in RTRs, particularly surrounding the feasibility and acceptability of high intensity interval training (HIIT) versus moderate intensity continuous training (MICT) in this population. This study aims to explore the feasibility of three different supervised aerobic exercise programmes in RTRs to guide the design of future large scale efficacy studies.

Methods and Analysis: Renal transplant recipients will be randomised to HIIT A (16 min interval training with 4, 2 and 1 minute intervals at 80-90% VO_2 peak), HIIT B (4 x 4 min interval training at 80-90% VO_2 peak) or MICT (~40 min cycling at 50-60% VO_2 peak) where they will undertake 24 supervised sessions (approximately thrice weekly over 8 weeks). Assessment visits will be at baseline, mid-training, immediate post-training and 3 months post-training. The study will evaluate the feasibility of recruitment, randomisation, retention, assessment procedures and the implementation of the interventions. A further qualitative sub-study (QPACE-KD) will explore patient experiences and perspectives through semi-structured interviews and focus groups.

Ethics and Dissemination: All required ethical and regulatory approvals have been obtained. Findings will be disseminated through conference presentations, public platforms and academic publications.

Trial Registration Number: Prospectively registered; ISRCTN17122775.

Strengths and limitations of this study
• This study provides a multidisciplinary approach with a variety of quantitative and qualitative outcome measures which will contribute to future large scale randomised control trial design with focus on negative CVD outcomes in RTRs<br>• This study addresses the urgent need to determine the feasibility of differing aerobic exercise types in RTRs to inform future advice and recommendations<br>• This study specifically contributes to the limited literature on the feasibility of high intensity interval training in solid organ transplant patients<br>• As a limitation, the sample size is limited due to the study being centred on feasibility in preparation for future efficacy trials

INTRODUCTION

Cardiovascular disease (CVD) is a major cause of morbidity and mortality after kidney transplantation and a key factor limiting graft survival.[1 2] Most recent data suggests that the proportion of mortalities in kidney transplant recipients attributed to CVD or stroke is 22%[3] however, this figure does not emphasise the total impact on graft survival and quality of life of non-fatal events such as acute myocardial infarction, heart failure, stroke and cardiac arrhythmias.[2] Numbers of transplants per year are currently rising,[3] highlighting the need for cost-effective CVD prevention and risk factor management strategies for sustained patient health, graft survival and reduced health care burden.[4]

Traditional models of predicting CVD risk underestimate the risk in chronic kidney disease (CKD) patients suggesting that kidney disease and transplantation introduce unique CVD risk factors not

For peer review only - http://bmjopen.bmj.com/site/about/guidelines.xhtml

akin to the general population.[5] Immunosuppressive medication can exacerbate traditional risk factors such as poor lipid profile, elevated blood pressure and the incidence of new onset diabetes.[6 7] Weight gain, predominantly fat, is common in RTRs with 60% having a mid-arm fat area that is within the top 10% of values for the general population.[8] Unique haemodynamic challenges (anaemia, hypertension and volume expansion) in RTRs can accelerate cardiomyopathy in the absence of the usual observation of concurrent ischemic heart disease.[2] These haemodynamic stresses are a likely contributor to the paradoxically common vascular and systemic inflammation observed in RTRs.[9] Studies have suggested that elevations in novel biomarkers of inflammation are not just indicative but also contributory to CVD development.[10 11]

Empirical evidence suggests that physical activity has ‘anti-inflammatory’ consequences.[11] Recent exercise intervention studies in kidney patients who have not reached end-stage renal disease (ESRD) demonstrate reduced traditional and novel markers of CVD risk and improved physical function.[12-14] Similarly, dialysis patients have shown lowered markers of systemic inflammation[15] and improvements in arterial stiffness.[16] However, given its probable benefits, there is a shortfall in literature surrounding the role of physical activity in the management of CVD risk in RTRs.[17] With a precarious balance of immune function and non-traditional CVD risk factors, current exercise guidelines may not be appropriate for this population.

Moderate intensity continuous training (MICT) such as 30-40 minutes brisk walking on three days of the week[12] or intradialytic cycling[15] have been utilised and shown to be beneficial in reducing markers of CVD risk in kidney disease patients. There has been a recent abundance of literature surrounding high intensity aerobic protocols, particularly high intensity intermittent training (HIIT) which suggests a greater improvement in traditional and inflammatory markers of CVD risk in clinical and non-clinical populations when compared to MICT.[18] Although protocols differ, HIIT is characterised by short bursts of vigorous exercise (upwards of 80% maximal aerobic capacity) interspersed with periods of moderate intensity exercise or rest. In patients with CVD and heart transplant recipients, findings indicate that HIIT is well tolerated, safe and effective in improving CVD risk.[19-21] Most recently, HIIT has been shown to be effective in improving physical function, inflammation and quality of life in peritoneal dialysis patients[22] however, the effectiveness of HIIT versus MICT in RTRs has not yet, to our knowledge, been evaluated. With a lower time commitment and the aforementioned superior benefits, HIIT has the potentially to provide a creditable alternative to MICT. This type of exercise has also been perceived to be more enjoyable than MICT,[23] a key component for changing and maintaining physical activity levels in RTRs identified through qualitative methods.[24] However, our patients' views suggest that although HIIT may provide superior improvements of CVD risk factors with a shorter time commitment, the strenuous nature of the exercise might in itself present a barrier to participation in this population[24], and MICT such as brisk walking, may be more acceptable and encourage sustained participation.

Considering both the development of effective and sustainable exercise guidelines for lowering the CVD risk in RTRs and producing programmes with a wide appeal and good adherence, this pilot research aims to generate substantial foundations towards understanding the feasibility and acceptability of differing aerobic exercise protocols in this unique population. The finding will contribute to the growing body of information not only in this population but in other chronic disease populations at risk of CVD and also determine the feasibility of proceeding to large and complex randomised control trials with primary outcomes focusing on the reduction of negative CVD outcomes.

Objectives

Primary Objective

For peer review only - http://bmjopen.bmj.com/site/about/guidelines.xhtml

To evaluate the feasibility of a trial protocol involving three different supervised aerobic exercise programmes in renal transplant recipients.

Secondary Objectives

1. To assess participant experience and acceptability.
2. To explore potential for impact on physical function, body composition, habitual physical activity and non-invasive CVD risk factors.
3. To explore potential for impact on relevant blood, saliva and urinary markers of metabolic health, systemic inflammatory status and immune function.
4. To assess the tolerability, practicality and patient perceived usefulness of these assessments of cardiovascular disease risk.
5. To explore patient perception of their illness and symptoms, activity levels and quality of life.

METHODS AND ANALYSIS

PACE-KD Trial Design

The PACE-KD trial is a randomised, three-arm parallel-group study to determine the feasibility and acceptability of three different supervised aerobic exercise programmes. Figure 1 presents the study flow chart. There will be three parallel groups (exercise programmes): HIIT intervention A, HIIT intervention B and MICT (detailed below). Participants will be randomised to one of the three study arms and will complete 24 supervised sessions of the intervention (approximately three sessions per week over eight weeks). After initial baseline testing, outcome measures will be attained at mid-training, immediate post training and three months post training. Additional acute outcome measures will be taken during one exercise session in the first six weeks and during one exercise session in the last six weeks of each exercise programme (See Figure 2). Feasibility will be determined by assessing recruitment, compliance and completion. Acceptability and patient experience will be determined by a Patient Satisfaction Questionnaire (PSQ) and an optional nested qualitative study (QPACE-KD) consisting of either one to one semi-structured interviews or focus groups.

Patient Involvement

The study was designed in consultation with patient partners. A patient involvement (PPI) group has been convened (lead by JS) and will meet regularly with the rest of the research team to review progress and address any issues that arise. The PPI partners will particularly take part in the qualitative analysis, and interpretation and dissemination of the results.

Participants and Recruitment

A total of 24 participants with an established kidney transplant will be randomised (via computerised random number generator) to either HIIT intervention A, HIIT intervention B or MICT (see Table 1 for detailed inclusion and exclusion criteria). Patients will be screened by their own clinician for eligibility to enter the study. Eligible patients will be approached during their routine clinical appointments and will be provided with verbal and written study information. Additionally, eligible patients who have given prior consent to be contacted regarding research opportunities will be contacted via the post. All patients will be given the opportunity to discuss the study in more detail and the opportunity to consider their participation (at least 48 hours).

For peer review only - http://bmjopen.bmj.com/site/about/guidelines.xhtml

Table 1. PACE-KD Inclusion and Exclusion Criteria	
**Inclusion Criteria**	**Exclusion Criteria**
• Diagnosed with CKD and are a renal transplant recipient<br>• Male or female, aged 18 years or over<br>• Received renal transplant > 12 weeks prior to entering the study<br>• Is willing and able to give informed consent for participation in the study	• Aged under 18 years<br>• Female participants who are pregnant, lactating, or planning pregnancy during the course of the study<br>• Scheduled elective surgery or other procedures requiring general anaesthesia during the study<br>• Any other significant disease or disorder*<br>• Inability to give informed consent or comply with testing and training protocol for any reason

*i.e. significant co-morbidity including unstable hypertension, potentially lethal arrhythmia, myocardial infarction within 6 months, unstable angina, active liver disease, uncontrolled diabetes mellitus (HbA1c ≥ 9%), advanced cerebral or peripheral vascular disease which, in the opinion of the patient's own clinician, may either put the patient at risk because of participation in the study, or may influence the result of the study, or the patient's ability to participate in the study.

Interventions

Each exercise programme will consist of 24 sessions over approximately 8 weeks. Participants will be allowed to drink plain water during exercise sessions. The programmes will be based on the three protocols described below.

HIIT programme A: (Figure 3a)

Each session will consist of 16-min interval training with intervals of 4, 2 and 1 min duration at 80%, progressing to 90% $VO_{2\ peak}$ over the 8 weeks, separated by a 2 min active rest (~60% $VO_{2\ peak}$) giving a total exercise time of 30 min.

HIIT programme B: (Figure 3b)

Each supervised session will consist of 4 x 4-min of interval training at 80% $VO_{2\ peak}$, progressing to 90% $VO_{2\ peak}$ over the 8 weeks, separated by a 3 min active rest (~60% $VO_{2\ peak}$) and with a final 5 min recovery stage to ensure that overall work done is matched for both HIIT protocols, therefore also giving a total exercise time of 30 min.

MICT Exercise Intervention

Each supervised session will consist of continuous brisk cycling for ~40 min with a target RPE of 12-14 (somewhat hard). This is equivalent to 50-60% $VO_{2\ peak}$ and will be matched to the HIIT protocols for total external work done based on calculations of percentage of peak watts obtained from the $VO_{2\ peak}$ test. In this way, for each individual the overall energy expenditure during the session would be the same regardless of the trial they are assigned to.

All exercise sessions will be preceded by a 5 min low intensity warm up and followed by a 10 min cool down.

For peer review only - http://bmjopen.bmj.com/site/about/guidelines.xhtml

Study Procedures

The study outcomes, measurement methods and assessment time periods are summarised in Table 2 and a detailed overview of each measurement is provided at the end of this section. Data on recruitment, numbers of sessions attended and completed in accordance with the protocol and drop out will be recorded and continuously monitored.

Table 2. PACE-KD outcomes, measurement methods and assessment time periods

Outcome	Measurement Method/Tool	Time Period
Feasibility	Recruitment, compliance, drop-out and completion percentages	Continuous
$VO_{2\ peak}$	CPET	Baseline, post-training
Physical Function	Gait speed test<br>Sit to stand test<br>Plantar flexion Strength (Fysiometer)	Baseline, mid-training, post-training, 3M post-training
Body Composition	BIA	Baseline, mid-training, post-training, 3M post-training
Heart Function	NICOM	Baseline, mid-training, post-training, 3M post-training
Arterial Stiffness	PWV (Applanation Tonometry)	Baseline, mid-training, post-training, 3M post-training
SpO_2	Pulse Oximetry	Baseline, mid-training, post-training, 3M post-training, acute response sessions
Habitual Physical Activity	Accelerometry	Baseline, post-training
Muscle O_2 Saturation	NIRS	Acute response session
Urinary Bacteriuria	Urine Sample	Baseline, mid-training, post-training
Immune Function	Blood and Saliva Samples	Baseline, mid-training, post-training, 3M post-training, acute response sessions
Markers of inflammation, oxidative stress, CVD risk	Blood Samples	Baseline, mid-training, post-training, 3M post-training, acute response sessions
Renal Profile	Blood Samples	Baseline, post training
Clinical Information	Extraction from clinical notes	Baseline, mid-training, post-training, 3M post-training
Renal Symptoms	POS-S	Baseline, mid-training, post-training, 3M post-training
Fatigue	Fatigue Scale	Baseline, mid-training, post-training, 3M post-training
Quality of Life	EQ-5D	Baseline, mid-training, post-training, 3M post-training
Illness Perception	IPQ-R	Baseline, mid-training, post-training, 3M post-training
Physical Activity/ Functional Capacity	DASI	Baseline, mid-training, post-training, 3M post-training
Pain	BPI	Baseline, mid-training, post-training, 3M post-training
Sleep Quality	PSQI	Baseline, mid-training, post-training, 3M post-training
Daytime Sleepiness	ESS	Baseline, mid-training, post-training, 3M post-training
Illness Symptoms	PACE-KD Illness-Symptom Questionnaire	Before each exercise session and x1 at the weekend between sessions
Tolerability, practicality, acceptability and perceived usefulness of the study measures	PSQ;<br>Semi-structured interview or focus group	Post study involvement;<br>Post study involvement through optional QPACE-KD nested qualitative study

Abbreviations: $VO_{2\ peak}$, Peak Oxygen Uptake; CPET, Cardiopulmonary Exercise Test; BIA, Bioelectrical Impedance Analysis; NICOM, Non-Invasive Cardiac Output Monitor; SpO_2, Peripheral Oxygen Saturation; NIRS, Near-infrared Spectroscopy; CVD, Cardiovascular Disease; POS-S, Renal Patient Outcome Scale; EQ-5D, EuroQol; IPQ-R, Illness Perception Questionnaire - Revised; DASI, Duke Activity Status Index; BPI, Brief Pain Inventory; PSQI, Pittsburgh Sleep Quality Index; ESS, Epworth Sleepiness Scale; PSQ, Patient Satisfaction Questionnaire

Baseline Visit (Visit 1)

At visit one informed consent will be obtained on arrival. Participants will perform a standard maximal exercise test ($VO_{2\ peak}$ test) in the presence of a cardiac nurse. This test will be preceded by a 12-lead electrocardiogram (ECG) and subject to the confirmed absence of any contraindications.

For peer review only - http://bmjopen.bmj.com/site/about/guidelines.xhtml

Measurements of cardiac function, physical function, anthropometry, body composition and blood, urine and saliva samples will be collected. A survey pack will be completed and an accelerometer given to measure habitual physical activity over approximately 7 days.

Training visits

Participants will attend 24 supervised exercise sessions (approximately 3 per week for 8 weeks) of either: HIIT A, HIIT B or MICT (as outlined above). At the beginning of each session participants will be asked to complete an internally designed Illness-Symptom questionnaire. They will also be provided with a copy to complete over the weekend between sessions. The questionnaire will provide data on illness symptoms particularly relating to upper respiratory, digestive and urinary tracts.

Acute Response Sessions

At one of the first six and at one of the final six exercise sessions, a resting blood sample will be collected immediately prior to the usual exercise session, immediately post exercise session, 1 h post exercise session, 4 h post exercise session and immediately prior to the start of the next exercise session. Saliva samples will be taken in conjunction with each blood sample. Participants will be asked to abstain from caffeine and alcohol for 24 h prior to these two acute testing sessions, and on acute testing session 2 asked to consume a similar diet to the 24 h prior to acute session 1. During the exercise session a muscle oxygen saturation monitor will be attached to the calf muscle (non-invasively) and will be removed following the 4 h blood sample. Following the 1 h samples, prior to the 4 h sample, patients will be provided with light refreshments. They will be asked to refrain from consuming alcohol and caffeine and from partaking in strenuous exercise.

Mid-training Visit

The mid-training visit will take place between weeks 3 and 5. At this visit, the experimental tests described in the baseline visit will be repeated with the exception of the $VO_{2\ peak}$ test and the accelerometer for habitual physical activity. The survey pack as described in the baseline visit will be administered, completed and collected during this visit.

Immediate Post-training visit

The immediate post-training visit will take place within 14 days of the final exercise session taking place. All experimental tests and the survey pack outlined in the baseline visit will be repeated. An accelerometer will be fitted as in the baseline visit and returned after one week.

Three Month Post-Training Visit

The three month post-training visit will take place approximately three months following the final exercise session. The visit will be very similar to the mid-training visit. All experimental tests and the survey pack outlined in the aforementioned visit will be repeated with the exclusion of the $VO_{2\ peak}$ test. There will be no more experimental measures after this visit.

An internally designed Patient Satisfaction Questionnaire (PSQ) will be administered once at the end of study participation. The PSQ is designed to assess the acceptability of the study and experiences of the patients in each of the trial groups with particular focus on tolerability, practicality, enjoyment and perceived usefulness of the study measures and of the study as a whole. Participants who cease

For peer review only - http://bmjopen.bmj.com/site/about/guidelines.xhtml

involvement in the study prior the final assessments will also be invited to complete the questionnaire. This will be optional.

Study Outcome Measures

$VO_{2\ peak}$ Test

Standard ramp protocol; increasing workload of approximately 1 Watt every 4 sec (10-15 w/min) ensuring volitional exhaustion within 12-15 min[25] on a stationary electronically braked cycle ergometer. Participants will be encouraged to cycle at a continuous cadence (~70 rpm). The highest oxygen uptake will be measured in this study ($VO_{2\ peak}$) as true maximal oxygen uptake ($VO_{2\ max}$) is less commonly achieved in deconditioned and/or clinical patients. Results of this test will be used to determine training workloads.

Clinical information

Clinical information will be extracted from the medical notes including: age, gender, ethnicity, primary cause of renal failure, transplant type, time since transplant, dialysis duration, co-morbidities, blood/urine results, current medication and smoking habits. This information will be used to primarily account for cofounding variables and analyses of differences and similarities between intervention arm groups.

Anthropometric measures

Anthropometric measures of height, weight and waist and hip circumference will be attained in accordance with standard protocols.[26]

Body Composition

Bioelectrical impedance analysis (BIA) performed on an InBody analyser (InBody 370, Chicago, IL, USA) will be used to assess body composition (e.g. body fat percentage and fat free mass). This is a painless, non-invasive method[27 28] previously validated for use in CKD patients.[29]

Cardiovascular Condition and Function

A Non-Invasive Cardiac Output Monitor (NICOM; Cheetah Medical, Maidenhead, UK) will be used to assess heart function using cardiac bioreactance analysis. This method is safe, quick and validated[30] for assessing haemodynamic parameters including cardiac output (heart rate x stroke volume) and total peripheral resistance.

Pulse Wave Analysis (PWA)

Pulse wave analysis will be measured non-invasively to determine arterial stiffness using applanation tonometry methods (Sphygmocor, AtCor Medical Pty Ltd, West Ryde, NSW, Australia). Arterial pressure waveforms are recorded superficially at the radial artery by an experienced laboratory technician by applanating the artery with a high-fidelity hand-held tonometer at the site of maximal pulsation. Consistent and reproducible waveforms are recorded for 20 seconds. This is a safe procedure and a measure of this type has been performed in CKD patients previously.[13 14]

Physical Function

Physical function will be assessed using three tests. The Sit-to-stand (STS) test will be used to ascertained functional ability and has been widely used in the CKD population.[31-33] Participants will

For peer review only - http://bmjopen.bmj.com/site/about/guidelines.xhtml

be asked to sit on a hard upright chair with their feet flat on the floor and knees bent at 90°. One cycle is considered complete when the participant stands up and sits down again to the starting position (without using their hands). There are two tests within the STS:

- *STS5*: The time taken for patients to stand up from a seated position and sit back down again 5 times. It is a surrogate measure of muscular power.

- *STS60* : The number of STS cycles achieved in 60 seconds and is a surrogate measure of muscular endurance.

The 4 meter walk test will be used to assess gait speed. Participants will be asked to walk 4 meters at their usual pace for one practice and two times trials. The better score (in sec) on the timed trials will be recorded.

Isometric plantar flexion strength (gastrocnemius and soleus strength) will be assessed using a Wii Fit board (Nintendo, Kyoto, Japan). Participants will sit with their legs bent to 90° with their feet flat on the board. A strap will be placed around the board and over the knees. Participants will then be asked to raise their heels off the board causing a downward force to be exerted on the board.

Muscle Oxygen Saturation

Prior to the acute testing sessions a small non-invasive muscle oxygen saturation device (BSXInsight, BSXAthletics, TX, USA) will be placed onto the calf muscle and secured with a small bandage. The device will be removed following the 4 h sample collection. This will measure muscle oxygen saturation using near infrared spectroscopy (NIRS) and has been used in a variety of diseases previously.[34]

Saliva sampling and storage

Saliva samples will be collected into sterile plastic containers. Participants will swallow to empty the mouth, then open and hold the container themselves before performing a passive dribble of saliva collected under the tongue over the next two minutes. Following centrifugation, the supernatant will be aliquoted and frozen for future analysis, primarily for secretory Immunoglobulin A (sIgA) to investigate mucosal immunity. Urine Sampling and Storage

A urine sample will be requested during the baseline visit. This sample will be analysed for urinary protein excretion, bacteriuria and urinary tract infection susceptibility.

Venous Blood Sampling and Analysis

Venous blood will be collected using venepuncture of the antecubital vein and prepared appropriately for the following analysis:

- A cytometric bead array technique will be used to allow bulk-analysis of a panel of pro- and anti-inflammatory cytokines, including but not limited to IL-1, IL-2, IL-6, IL-10, TNF-α and IFNγ.
- Other markers of inflammation (CRP) and oxidative stress (MDA) and will be assessed by ELISA.
- Phenotyping of microparticles using flow cytometry.

For peer review only - http://bmjopen.bmj.com/site/about/guidelines.xhtml

- Immune cell subsets using flow cytometry.
- Full blood count and renal profile analysis, which includes eGFR, urea, bicarbonate, creatinine, sodium, potassium and phosphate measures.

Questionnaires

Participants will be asked to complete a survey pack containing the following: (1) Renal Patient Outcome Scale (POS-S); a validated questionnaire that measures the presence and severity of disease related symptoms with the addition of a transplant specific question set.[35] (2) Fatigue Scale; an internally designed Likert scale on feelings of tiredness. (3) EuroQol (EQ-5D); a short widely used quality of life questionnaire.[36] (4) Illness Perception Questionnaire – Revised (IPQ-R); a 84 item questionnaire which is used to assess the principle components of illness representations.[37] This questionnaire has been tailored for patients with chronic kidney disease and will assess their perceptions of physical activity as treatment. (5) Duke Activity Status Index (DASI); a 12-item questionnaire that utilizes self-reported physical work capacity to estimate peak metabolic equivalents (METs) and has been shown to be a valid measurement of functional capacity.[38] (6) Brief Pain Inventory – Short Form (BPI-sf); a 9 item self-administered questionnaire used to evaluate the severity of a patient's pain and the impact of this pain on the patient's daily functioning.[39]. (7) The Pittsburgh Sleep Quality Index (PSQI); a self-report questionnaire that assesses sleep quality over a 1-month time interval[40] (8) Epworth Sleepiness Scale (ESS); the ESS measures a person's general level of daytime sleepiness, or their average sleep propensity in daily life (ASP). [41]

Accelerometry

An ActiGraph wGT3X-BT accelerometer (Actigraph, Pensacola, Florida, USA) will be provided to wear on the waist which will record habitual physical activity (returned after one week).

Nested Qualitative Study (QPACE-KD)

Six participants from each intervention arm will be invited to an audio-recorded focus group or semi-structured interview to discuss their experiences of the study and the intervention in more detail and to gather suggestions for relevant and acceptable design of future randomised controlled trials. The qualitative phase will be conducted by a practiced researcher uninvolved with the aforementioned study protocol to encourage freedom of expression. Both one-to-one semi-structured interviews and focus groups will be used to provide different but complementary qualitative information. The interviews will ascertain individual perspectives and feelings about the intervention and outcome protocol in the light of personal and individual experiences of having completed the feasibility study. On the other hand, focus groups will elicit perceptions which are particularly useful when there is some consensus and meaning about the existence of a community or shared voice.

The QPACE-KD study is an optional addition to PACE-KD. The QPACE-KD protocol will be explained to the patient, and they will be given ample opportunity to ask questions and at least 48 hours to decide whether or not to participate. If they agree, separate QPACE-KD written informed consent will be attained before the interview or focus group takes place. During the interviews and focus groups, the facilitator will introduce the process, explaining the background and reasons for the study, how the interview will proceed, and details of audio-recording and note-taking. Participants will be reassured about the preservation of their anonymity and confidentiality as well as given the opportunity to ask questions.

Transcripts will only include non-identifying information. Individual's names and personal details will not be included in the completed transcripts. Similarly, any written notes taken during the interviews will not include any identifiable personal data.

For peer review only - http://bmjopen.bmj.com/site/about/guidelines.xhtml

Data Collection and Management

Data at all time points will be collected in case report forms by the trial team. All data will be entered into a secure database and will only be accessible on password protected computers at University Hospitals of Leicester NHS Trust, University of Leicester and Loughborough University by relevant members of the study team. No identifying information will be kept in electronic form. All source data and original participant identities will be kept in a locked office in the trial site file only at University Hospitals of Leicester NHS Trust.

Sample Size

As the trial has a primary outcome of feasibility the study sample size is pragmatic and a power calculation is neither relevant nor possible. Data collected on secondary outcome measures will provide data on which sample size calculations can be performed in future well informed randomised controlled trials. We have chosen specific and relevant secondary outcome variables that may be suitable future primary outcomes.

Data Analysis

The primary outcome will assess the feasibility of study components such as recruitment, randomisation, retention, assessment procedures and the implementation of the interventions. These will be quantified by analysing factors such as but not limited to: numbers screened, numbers enrolled, proportion of eligible patients enrolled, intervention-specific retention rates, rates of adherence to protocol per intervention and proportion of training sessions and assessment sessions completed. The criteria for determining study and intervention feasibility will be as follows: Compliance: attending >70% of sessions and Retention/completion: >80% of participants.

Due to the small sample size, complex statistical analysis will not be performed on the secondary outcomes; however, we will use simple analysis (eg. T-test, ANOVA) in order to investigate statistical differences for exploratory information only.

Interview data will be analysed according to the principles of interpretive thematic analysis using a framework approach[42] to explore themes emerging from patient journeys through and experiences of the interventions and outcome measures. The whole research team, including patient partners, will be involved in qualitative analysis to avoid individual bias.

Ethics and Dissemination

Ethical issues

The University Hospitals of Leicester NHS Trust (UHL) agreed to act as sponsor for this study on 31/10/2016 (EDGE 88714). The protocol was reviewed by the East Midlands-Nottingham Research Ethics Committee (REC) and was given a favourable opinion (REC ref 16/EM/0482) on 04/01/2017. Health Research Authority regulatory approval was given on 27/01/2017, and the study was adopted on the NIHR portfolio on 12/01/2017. Local governance approval was granted by UHL Research and Innovation on 02/03/2017. Steps have been taken when designing this protocol to minimise the ethical implications and ensure patient welfare. The study will comply with the International Conference for Harmonisation of Good Clinical Practice (ICH GCP) guidelines and the Research Governance Framework for Health and Social Care.

Dissemination Plan and Impact

For peer review only - http://bmjopen.bmj.com/site/about/guidelines.xhtml

On completion the results of this study will be published in peer-reviewed journals and presented at national and international conferences. Participant level data will be available following publication of results on request. Results will also be disseminated to the patient and public community via social media and newsletter articles and presentations at patient conferences and forums, lead by the patient partners.

It is anticipated that the results of this study will inform future design of larger randomised controlled trials in this subject area and contribute to future specific physical activity guidelines in this population.

Trial registration

This study was prospectively registered with the ISRCTN (ISRCTN17122775).

Twitter: Follow Roseanne Billany @RBillany

Contributors: ACS is the chief investigator for this trial. ACS and NCB lead on study design and protocol preparation. RB wrote this manuscript and contributed to protocol preparation, ethics application and document preparation. All authors reviewed and approved the final version of this manuscript.

Funding: This study is jointly funded by Heart Research UK grant number RG2650/15/18, the Stoneygate Trust and the Engineering and Physical Sciences Research Council (EPSRC) Antimicrobial Resistance (AMR) grant. The research was supported by the National Institute for Health Research (NIHR) Leicester Biomedical Research Centre. The views expressed are those of the authors and not necessarily those of the NHS, the NIHR or the Department of Health.

Acknowledgements: We would like to thank Dr Clare Stevinson (Loughborough University), Dr James O Burton (University Hospitals of Leicester NHS Trust) and Mr John Savage for their valuable work towards the development of the funding application for this study.

Competing Interests: None declared.

Figure legends:

Figure 1. Study Flowchart: Overall study diagram.

Figure 2. Study Flowchart: Acute response sessions detailed diagram.

Figure 3. Schematic diagram of HIIT regimens A and B. Each block shows stage duration in min. The final 5 minute stage has been added to HIIT programme B to ensure the overall work done is matched for both protocols.

References

1. Chan W, Bosch JA, Jones D, et al. Obesity in kidney transplantation. *J Ren Nutr* 2014;24(1):1-12.
2. Kiberd BA. Cardiovascular Disease in Kidney Transplant Recipients. *Adv Stud Med* 2007;7(6):169-78.

For peer review only - http://bmjopen.bmj.com/site/about/guidelines.xhtml

3. Pruthi R, Casula A, MacPhee I. UK Renal Registry 17th Annual Report: Chapter 3 Demographic and Biochemistry Profile of Kidney Transplant Recipients in the UK in 2013: National and Centre-specific Analyses. *Nephron* 2015;129 Suppl 1:57-86.
4. Mathur AK, Chang Y-H, Steidley ED, et al. Patterns of Care and Outcomes in Cardiovascular Disease After Kidney Transplantation in the United States. *Transplant Direct* 2017;3(2):e26.
5. Mansell H, Rosaasen N, Dean J, et al. Evidence of enhanced systemic inflammation in stable kidney transplant recipients with low Framingham risk scores. *Clin Transplant* 2013;27(4):E391-9.
6. Boots JM, Christiaans MH, van Hooff JP. Effect of immunosuppressive agents on long-term survival of renal transplant recipients: focus on the cardiovascular risk. *Drugs* 2004;64(18):2047-73.
7. Heisel O, Heisel R, Balshaw R, et al. New onset diabetes mellitus in patients receiving calcineurin inhibitors: a systematic review and meta-analysis. *Am J Transplant* 2004;4(4):583-95.
8. Schutz T, Hudjetz H, Roske AE, et al. Weight gain in long-term survivors of kidney or liver transplantation - another paradigm of sarcopenic obesity? *Nutrition* 2012;28(4):378-83.
9. Abedini S, Holme I, Marz W, et al. Inflammation in renal transplantation. *Clin J Am Soc Nephrol* 2009;4(7):1246-54.
10. Kasapis C, Thompson PD. The effects of physical activity on serum C-reactive protein and inflammatory markers: a systematic review. *J Am Coll Cardiol* 2005;45(10):1563-69.
11. Gleeson M, Bishop NC, Stensel DJ, et al. The anti-inflammatory effects of exercise: mechanisms and implications for the prevention and treatment of disease. *Nat Rev Immunol* 2011;11(9):607-15.
12. Viana JL, Kosmadakis GC, Watson EL, et al. Evidence for anti-inflammatory effects of exercise in CKD. *J Am Soc Nephrol* 2014;25(9):2121-30.
13. Mustata S, Groeneveld S, Davidson W, et al. Effects of exercise training on physical impairment, arterial stiffness and health-related quality of life in patients with chronic kidney disease: a pilot study. *Int J Nephrol Urol* 2011;43(4):1133-41.
14. Kosmadakis GC, John SG, Clapp EL, et al. Benefits of regular walking exercise in advanced pre-dialysis chronic kidney disease. *Nephrol Dial Transplant* 2012;27(3):997-1004.
15. Dungey M, Bishop NC, Young HML, et al. A Six-Month Intradialytic Exercise Programme Has Anti-Inflammatory Effects on Circulating Monocyte Phenotypes and Regulatory T Cells But Not on Cytokine Concentration. 52nd ERA-EDTA Congress. London, 2015.
16. Mustata S, Chan C, Lai V, et al. Impact of an exercise program on arterial stiffness and insulin resistance in hemodialysis patients. *J Am Soc Nephrol* 2004;15(10):2713-18.
17. Didsbury M, McGee RG, Tong A, et al. Exercise training in solid organ transplant recipients: a systematic review and meta-analysis. *Transplantation* 2013;95(5):679-87.
18. Ramos JS, Dalleck LC, Tjonna AE, et al. The impact of high-intensity interval training versus moderate-intensity continuous training on vascular function: a systematic review and meta-analysis. *Sports Med* 2015;45(5):679-92.
19. Rognmo O, Hetland E, Helgerud J, et al. High intensity aerobic interval exercise is superior to moderate intensity exercise for increasing aerobic capacity in patients with coronary artery disease. *Eur J Cardiovasc Prev Rehabil* 2004;11(3):216-22.
20. Wisloff U, Stoylen A, Loennechen JP, et al. Superior cardiovascular effect of aerobic interval training versus moderate continuous training in heart failure patients: a randomized study. *Circulation* 2007;115(24):3086-94.
21. Dall CH, Gustafsson F, Christensen SB, et al. Effect of moderate- versus high-intensity exercise on vascular function, biomarkers and quality of life in heart transplant recipients: A randomized, crossover trial. *J Heart Lung Transplant* 2015;34(8):1033-41.
22. Rouchon MI, Coutard C, Matysiak M et al. High Intensity Interval Training Improves Physical Functioning, Inflammation and Quality of Life in Peritoneal Dialysis Patients. *Nephrol Dial Transplant* 2016;31(suppl_1)

For peer review only

For peer review only - http://bmjopen.bmj.com/site/about/guidelines.xhtml

23. Bartlett JD, Close GL, MacLaren DP, et al. High-intensity interval running is perceived to be more enjoyable than moderate-intensity continuous exercise: implications for exercise adherence. *J Sports Sci* 2011;29(6):547-53.
24. Lloyd-Davies LH, Clarke AL, Brown SA, et al. Renal Transplant Recipient Perspectives on Physical Activity and Exercise. Proceedings of the American Society of Nephrology Annual Meeting 2014.
25. Ross R. ATS/ACCP Statement on Cardiopulmonary Exercise Testing. *Am J Respir Crit Care Med* 2003;167(2).
26. Eston R, Reilly T. Kinanthropometry and Exercise Physiology Laboratory Manual: Tests, Procedures and Data. 3 ed. Oxon: Routledge 2009.
27. Lukaski HC, Bolonchuk WW, Hall CB, et al. Validation of tetrapolar bioelectrical impedance method to assess human body composition. *J Appl Physiol* 1986;60(4):1327-32.
28. Macdonald JH, Marcora SM, Jibani M, et al. Bioelectrical impedance can be used to predict muscle mass and hence improve estimation of glomerular filtration rate in non-diabetic patients with chronic kidney disease. *Nephrol Dial Transplant* 2006;21(12):3481-87.
29. Chertow GM, Lowrie EG, Wilmore DW, et al. Nutritional assessment with bioelectrical impedance analysis in maintenance hemodialysis patients. *J Am Soc Nephrol* 1995;6(1):75-81.
30. Squara P, Denjean D, Estagnasie P, et al. Noninvasive cardiac output monitoring (NICOM): a clinical validation. *Intensive Care Med* 2007;33(7):1191-94.
31. Koufaki P, Mercer TH, Naish PF. Effects of exercise training on aerobic and functional capacity of end-stage renal disease patients. *Clin Physiol Funct Imaging* 2002;22(2):115-24.
32. McIntyre CW, Selby NM, Sigrist M, et al. Patients receiving maintenance dialysis have more severe functionally significant skeletal muscle wasting than patients with dialysis-independent chronic kidney disease. *Nephrol Dial Transplant* 2006;21(8):2210-16.
33. Johansen KL, Painter PL, Sakkas GK, et al. Effects of resistance exercise training and nandrolone decanoate on body composition and muscle function among patients who receive hemodialysis: A randomized, controlled trial. *J Am Soc Nephrol* 2006;17(8):2307-14.
34. Grassi B, Quaresima V. Near-infrared spectroscopy and skeletal muscle oxidative function in vivo in health and disease: a review from an exerise physiology perspective. *J Biomed Opt* 2016;21(9)
35. Hearn J, Higginson IJ. Development and validation of a core outcome measure for palliative care: the palliative care outcome scale. Palliative Care Core Audit Project Advisory Group. *Qual Health Care* 1999;8(4):219-27.
36. Herdman M, Gudex C, Lloyd A, et al. Development and preliminary testing of the new five-level version of EQ-5D (EQ-5D-5L). *Qual Life Res* 2011;20(10):1727-36.
37. Moss-Morris R. The Revised Illness Perception Questionnaire (IPQ-R). *Psychol Health* 2002;17(1):1-16.
38. Hlatky MA, Boineau RE, Higginbotham MB, et al. A brief self-administered questionnaire to determine functional capacity (the Duke Activity Status Index). *Am J Cardiol* 1989;64(10):651-54.
39. Cleeland CS, Ryan KM. Pain assessment: global use of the Brief Pain Inventory. *Ann Acad Med Singapore* 1994;23(2):129-38.
40. Buysse DJ, Reynolds CF, 3rd, Monk TH, et al. The Pittsburgh Sleep Quality Index: a new instrument for psychiatric practice and research. *Psychiatry Res* 1989;28(2):193-213.
41. Johns MW. A new method for measuring daytime sleepiness: the Epworth sleepiness scale. *Sleep* 1991;14(6):540-45.
42. Smith J, Firth J. Qualitative data analysis: the framework approach. *Nurse Res* 2011;18(2):52-62.

For peer review only

For peer review only - http://bmjopen.bmj.com/site/about/guidelines.xhtml

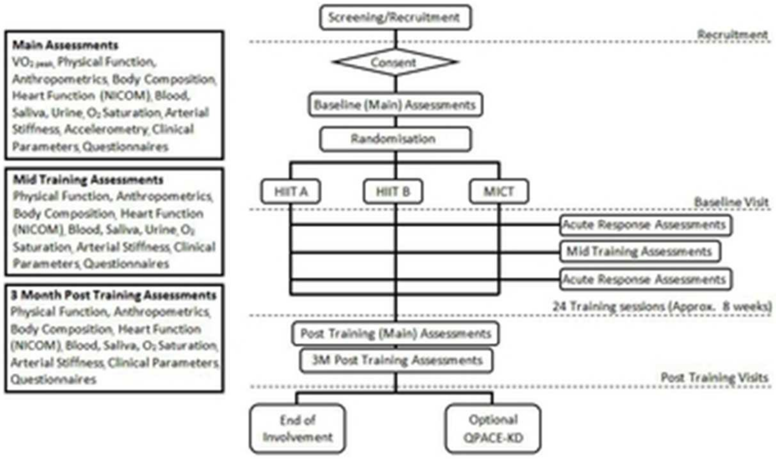

Figure 1. Study Flowchart: Overall study diagram.

35x20mm (300 x 300 DPI)

For peer review only

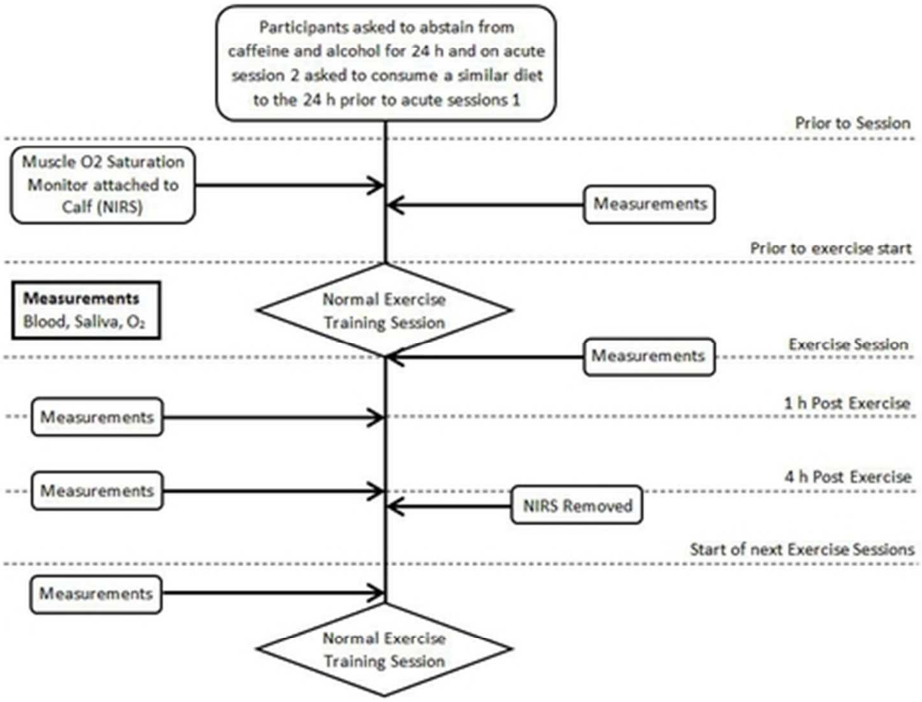

Figure 2. Study Flowchart: Acute response sessions detailed diagram.

43x33mm (300 x 300 DPI)

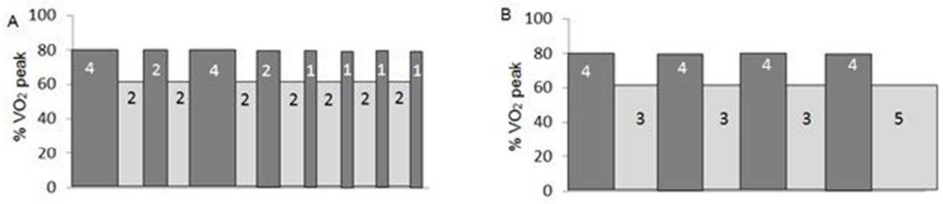

Figure 3. Schematic diagram of HIIT regimens A and B. Each block shows stage duration in min. The final 5 minute stage has been added to HIIT programme B to ensure the overall work done is matched for both protocols.

56x13mm (300 x 300 DPI)

For peer review only

CONSORT 2010 checklist of information to include when reporting a randomised trial*

Section/Topic	Item No	Checklist item	Reported on page No
**Title and abstract**			
	1a	Identification as a randomised trial in the title – not included at it is a feasibility study and protocol paper so space was limited.	x
	1b	Structured summary of trial design, methods, results, and conclusions (for specific guidance see CONSORT for abstracts)	2
**Introduction**			
Background and objectives	2a	Scientific background and explanation of rationale	2-3
	2b	Specific objectives or hypotheses	3-4
**Methods**			
Trial design	3a	Description of trial design (such as parallel, factorial) including allocation ratio	4
	3b	Important changes to methods after trial commencement (such as eligibility criteria), with reasons	N/A
Participants	4a	Eligibility criteria for participants	4-5
	4b	Settings and locations where the data were collected	4
Interventions	5	The interventions for each group with sufficient details to allow replication, including how and when they were actually administered	5
Outcomes	6a	Completely defined pre-specified primary and secondary outcome measures, including how and when they were assessed	6-10
	6b	Any changes to trial outcomes after the trial commenced, with reasons	N/A
Sample size	7a	How sample size was determined	11
	7b	When applicable, explanation of any interim analyses and stopping guidelines	N/A
Randomisation:			
Sequence generation	8a	Method used to generate the random allocation sequence	4
	8b	Type of randomisation; details of any restriction (such as blocking and block size)	4
Allocation concealment mechanism	9	Mechanism used to implement the random allocation sequence (such as sequentially numbered containers), describing any steps taken to conceal the sequence until interventions were assigned	
Implementation	10	Who generated the random allocation sequence, who enrolled participants, and who assigned participants to interventions	4

For peer review only

For peer review only - http://bmjopen.bmj.com/site/about/guidelines.xhtml

Blinding	11a	If done, who was blinded after assignment to interventions (for example, participants, care providers, those assessing outcomes) and how	N/A
	11b	If relevant, description of the similarity of interventions	5
Statistical methods	12a	Statistical methods used to compare groups for primary and secondary outcomes	11
	12b	Methods for additional analyses, such as subgroup analyses and adjusted analyses	11
**Results**			
Participant flow (a diagram is strongly recommended)	13a	For each group, the numbers of participants who were randomly assigned, received intended treatment, and were analysed for the primary outcome	N/A protocol
	13b	For each group, losses and exclusions after randomisation, together with reasons	N/A protocol
Recruitment	14a	Dates defining the periods of recruitment and follow-up	N/A protocol
	14b	Why the trial ended or was stopped	N/A protocol
Baseline data	15	A table showing baseline demographic and clinical characteristics for each group	N/A protocol
Numbers analysed	16	For each group, number of participants (denominator) included in each analysis and whether the analysis was by original assigned groups	N/A protocol
Outcomes and estimation	17a	For each primary and secondary outcome, results for each group, and the estimated effect size and its precision (such as 95% confidence interval)	N/A protocol
	17b	For binary outcomes, presentation of both absolute and relative effect sizes is recommended	N/A protocol
Ancillary analyses	18	Results of any other analyses performed, including subgroup analyses and adjusted analyses, distinguishing pre-specified from exploratory	N/A protocol
Harms	19	All important harms or unintended effects in each group (for specific guidance see CONSORT for harms)	N/A protocol
**Discussion**			
Limitations	20	Trial limitations, addressing sources of potential bias, imprecision, and, if relevant, multiplicity of analyses	N/A protocol
Generalisability	21	Generalisability (external validity, applicability) of the trial findings	N/A protocol
Interpretation	22	Interpretation consistent with results, balancing benefits and harms, and considering other relevant evidence	N/A protocol
**Other information**			
Registration	23	Registration number and name of trial registry	12
Protocol	24	Where the full trial protocol can be accessed, if available	N/A protocol
Funding	25	Sources of funding and other support (such as supply of drugs), role of funders	12

*We strongly recommend reading this statement in conjunction with the CONSORT 2010 Explanation and Elaboration for important clarifications on all the items. If relevant, we also recommend reading CONSORT extensions for cluster randomised trials, non-inferiority and equivalence trials, non-pharmacological treatments, herbal interventions, and pragmatic trials. Additional extensions are forthcoming: for those and for up to date references relevant to this checklist, see www.consort-statement.org.

For peer review only - http://bmjopen.bmj.com/site/about/guidelines.xhtml

For peer review only
